# Perspective: Opportunities for advancing aquatic invertebrate welfare

**DOI:** 10.3389/fvets.2022.973376

**Published:** 2022-11-15

**Authors:** Sarah J. Wahltinez, Nicole I. Stacy, Catherine A. Hadfield, Craig A. Harms, Gregory A. Lewbart, Alisa L. Newton, Elizabeth A. Nunamaker

**Affiliations:** ^1^Department of Comparative, Diagnostic, and Population Medicine, College of Veterinary Medicine, University of Florida, Gainesville, FL, United States; ^2^Seattle Aquarium, Seattle, WA, United States; ^3^Department of Clinical Sciences and Center for Marine Sciences and Technology, College of Veterinary Medicine, North Carolina State University, Morehead City, NC, United States; ^4^College of Veterinary Medicine, North Carolina State University, Raleigh, NC, United States; ^5^ZooQuatic Laboratory, LLC, Baltimore, MD, United States; ^6^OCEARCH, Park City, UT, United States; ^7^Global Animal Welfare and Training, Charles River Laboratory, Wilmington, MA, United States

**Keywords:** anesthesia, animal welfare, euthanasia, humane slaughter, refinement

## Abstract

Welfare considerations and regulations for invertebrates have lagged behind those for vertebrates, despite invertebrates comprising more than 95% of earth's species. Humans interact with and use aquatic invertebrates for exhibition in zoos and aquaria, as pets, research subjects, and important food sources. Recent research has indicated that aquatic invertebrates, in particular cephalopod mollusks and decapod crustaceans, experience stress and may be able to feel pain. With this article, we present results of a survey on attitudes of aquatic animal health professionals toward aquatic invertebrate welfare and provide practical recommendations for advancing aquatic invertebrate welfare across four areas of opportunity: use of anesthesia, analgesia, and euthanasia; development of less invasive diagnostic and research sampling methods based on 3R principles; use of humane slaughter methods for aquatic invertebrates; and reducing impacts of invasive procedures in aquaculture and fisheries. We encourage consideration of these opportunities to achieve far-reaching improvements in aquatic invertebrate welfare.

## Introduction

Despite comprising >95% of the animal species on Earth (1), attention to invertebrate animal welfare has lagged behind those for vertebrate animals. Although the interest in ethics and anesthesia related to vertebrate animal welfare has been increasing since the mid-twentieth century (2, 3), invertebrates have been less in the focus of welfare research and regulations. Aquatic invertebrates are displayed in zoos and aquaria, kept as pets, used as research animals, and serve as food sources for humans and other animals. Efforts to provide high quality care, to improve public perception and trust, and to extend ethical responsibilities to all veterinary patients and research subjects have driven the need to be mindful of aquatic invertebrate welfare.

Complicating our ability to discuss aquatic invertebrate welfare is the variable complexity of aquatic invertebrate nervous systems—from sponges which lack true nervous tissues (4) to cephalopods with roughly half a billion neurons (5). Cephalopods and decapod crustaceans are considered “advanced invertebrates” and have been the focus of the majority of research regarding invertebrate welfare. Cephalopods (e.g., cuttlefish, nautilus, octopus, and squid) have arguably the most complex nervous system found in invertebrates (6) and have a large body of literature devoted to exploring their nociceptive capabilities and pain perception (7–11). Decapod crustaceans (e.g., prawn, crab, lobster, and crayfish) have also been the subject of similar studies on nociceptive capabilities and pain perception (12–14) as well as indicators of stress (15–18). Less information is available for other taxa. Given the evidence of pain perception and stress in aquatic invertebrates, welfare considerations provide opportunities for advancements. In addition to the opportunities discussed in this paper, welfare can be improved through further minimization of stressors and the provision of species-appropriate housing, diet, water quality, social structure, and choices within an enriched environment, where appropriate (19–22).

Legal protections for aquatic invertebrates vary by country and whether the animals are used for research or human consumption. Cephalopods in research are protected in the European Union by Directive 2010/63/EU (23); decapod crustaceans were recommended for inclusion in this legislation (24) but were ultimately not included. This legal protection does not extend to animals intended for human consumption. Cephalopods and decapod crustaceans are protected in Switzerland (25), Norway (26), and New Zealand (27). Octopus are protected in the UK (28), although a recent publication by the London School of Economics reported strong evidence of sentience in cephalopod mollusks and decapod crustaceans (29). In Canada, cephalopods and “some other higher invertebrates” are protected (30). In the United States, invertebrates are not included in the Animal Welfare Act (31) but may be included for oversight by certain Institutional Animal Care and Use Committees if requested by the funding agency.

Here we present and discuss a survey on attitudes of aquatic animal health professionals toward aquatic invertebrate welfare and then provide practical recommendations for advancing aquatic invertebrate welfare across four areas of opportunity.

## Current attitudes toward aquatic invertebrate welfare among aquatic animal health professionals

In November 2019, a 10-question informal, anonymous survey was distributed electronically to three veterinary medicine-focused professional email listservs to determine the attitudes of aquatic animal health professionals toward the welfare of aquatic invertebrates. The majority of the 112 respondents identified as veterinarians (87%) while others identified as animal care staff, pathologists, researchers, veterinary technicians or assistants, or veterinary students. Sixty-seven of 111 (60%) thought that invertebrates can feel pain and 52 of 61 (85%) thought that cephalopod mollusks could feel pain. Only 49% had attempted pain control in invertebrates. Seventy-five of 112 (67%) indicated that they strongly consider the welfare of the invertebrates when performing treatments, procedures, or euthanasia. Respondents indicated that they euthanized aquatic invertebrates most frequently due to illness (95%), followed by population control (20%), cosmetic reasons (15%), research (5%), diagnostics (2%), feed for other animals (2%), age-related reasons (2%), and health surveillance (1%). The most common methods for euthanasia, either individually or in combination, included immersion in tricaine methanesulfonate, otherwise known as MS-222 (58%), magnesium salts (52%), physical methods (30%), freezing (20%), immersion in alcohol (18%), and/or Aqui-S/clove oil/eugenol (13%). Less common methods included sodium pentobarbital (5%), removal from water (4%), isoflurane (2%), formalin (2%), 2-phenoxyethanol (1%), lidocaine (1%), propofol (1%), and “shock” (1%) which was not further defined. Fifty of 92 (54%) identified that they used a two-step process. The results of this survey highlighted the need for development and implementation of evidence-based guidelines to improve the welfare of aquatic invertebrates in various settings and as appropriate. Future research on the topic could benefit from a formal survey with more participants to enable further statistical analyses.

## Opportunity 1: Promote the use of anesthesia, analgesia, and euthanasia

Anesthesia, analgesia, and euthanasia can provide great improvements to aquatic invertebrate welfare when appropriately implemented. Anesthesia can be used to immobilize aquatic invertebrates for physical examination, sample collection and procedures, and to reduce stress and the potential for injury for both animal and handler. Commonly used anesthetic concentrations have been previously reported for a limited number of species (32–36). The selection of anesthetic should be based on a knowledge of species biology, mechanisms of action of the agent, clinical judgment and if possible, recent literature, though even published methods should be critically evaluated. While hypothermia, carbon dioxide, and calcium-free seawater have been utilized as anesthetics, these procedures likely induce physiologic derangements, and their use may raise welfare concerns. Anesthesia for aquatic invertebrates typically involves immersing the animal in a solution (such as magnesium salts or 1–10% ethanol) or providing flow of anesthetic solution across the animal. Care should be taken that the solution is at the same temperature, pH, and osmolality as the animal's life support system water and is aerated to prevent hypoxia, and that water quality is monitored during prolonged procedures. Invertebrates should be frequently monitored and adjustments to the concentration of anesthetic made to maintain an optimal anesthetic plane. While MS-222 is commonly used in aquatic animal medicine, it may not be the best anesthetic choice for some invertebrate taxa as high concentrations are required which may lead to substantial changes in water chemistry that potentially impact animal health (37, 38).

When performing invasive or potentially painful procedures, the use of analgesic medications should be considered. However, there is a lack of information on appropriate analgesic medications for aquatic invertebrates. The few published research studies have focused on the use of local anesthetics such as lidocaine, given the conservation of sodium channels across species (39, 40). Lidocaine injections appear to have analgesic properties in cephalopods due to blocked afferent nerve signals and the prevention of behavioral responses to noxious stimuli (8, 11). Topical benzocaine decreased behavioral responses of glass prawn (*Palaemon elegans*) to noxious stimuli, also indicating potential analgesia (41). While morphine has been frequently used in decapod crustacean research, the observed results appear to be from sedation rather than analgesic properties (42). More evidence-based analgesic options are needed for all taxa of aquatic invertebrates.

Euthanasia is used to describe the act of ending the life of an animal in a manner that minimizes or eliminates pain and distress. Slaughter on the other hand is the act of killing animals for human or another animal's consumption (43) and is discussed in Opportunity 3. A follow-up anonymous survey was distributed electronically in May 2022 to four professional email listservs, predominantly aquatic animal veterinarians, to determine current euthanasia techniques for aquatic invertebrates. Of the 36 respondents who had euthanized an aquatic invertebrate in the previous 2 years, 92% identified as clinical veterinarians. The results of the survey are reported in Table 1.

**Table 1:** Responses from an electronically delivered survey on currently used methods for euthanasia of aquatic invertebrates.

		**Taxa**					
**Solo step** **method**		**Bivalve and gastropod mollusks**	**Cephalopod mollusks**	**Cnidarians**	**Crustaceans**	**Echinoderms**	**Horseshoe crabs**
	Number of respondents (n)	12	23	24	27	21	13
	Single/solo step performed	5 (41.7%)	4 (17.4%)	9 (37.5%)	8 (29.6%)	6 (28.6%)	4 (30.8%)
Immersion	MS-222	2 (16.7%)	1 (4.3%)	1 (4.2%)	3 (11.1%)	1 (4.8%)	0
	Clove oil/Eugenol/AquiS^®^	0	1 (4.3%)	1 (4.2%)	2 (7.4%)	0	0
	Magnesium chloride or magnesium sulfate	2 (16.7%)	2 (8.7%)	5 (20.8%)	2 (7.4%)	4 (19.0%)	0
	1–10% Ethanol	0	0	0	1 (3.7%)	1 (4.8%)	0
	>50% Ethanol	0	0	0	2 (7.4%)	0	0
	Formalin	0	0	0	0	0	0
	2-PE	0	0	0	0	0	0
Injection	KCl	0	0	0	0	0	0
	Pentobarbital	0	0	0	1 (3.7%)	0	4 (30.8%)
	Lidocaine	0	0	0	0	0	0
	Propofol	0	0	0	0	0	0
	Physical method	0	0	1 (4.2%)	0	0	0
	Freezing	1 (8.3%)	1 (4.3%)	1 (4.2%)	1 (3.7%)	0	0
	Removal from water	0	0	0	0	0	0
	Other	0	0	0	1 (3.7%)	0	0
		**Taxa**					
**1**^st^ **step** **method**		**Bivalve and gastropod** **mollusks**	**Cephalopod** **mollusks**	**Cnidarians**	**Crustaceans**	**Echinoderms**	**Horseshoe** **crabs**
Immersion	MS-222	0	1 (4.3%)	5 (20.8%)	4 (14.8%)	3 (14.3%)	1 (7.7%)
	Clove oil/Eugenol/AquiS^®^	0	1 (4.3%)	1 (4.2%)	5 (18.5%)	2 (9.5%)	2 (15.4%)
	Magnesium chloride or magnesium sulfate	6 (50%)	10 (43.5%)	9 (37.5%)	7 (25.9%)	10(47.6%)	3 (23.1%)
	1–10% Ethanol	3 (25%)	7 (30.4%)	4 (16.7%)	2 (7.4%)	3 (14.3%)	0
	>50% Ethanol	1 (8.3%)	5 (21.7%)	2 (8.3%)	0	2 (9.5%)	0
	Formalin	0	0	1 (4.2%)	0	0	0
	2-PE	0	0	0	1 (3.7%)	1 (4.8%)	3 (23.1%)
Injection	KCl	0	0	0	3 (11.1%)	0	1 (7.7%)
	Pentobarbital	0	1 (4.3%)	0	2 (7.4%)	1 (4.8%)	1 (7.7%)
	Lidocaine	0	0	0	0	0	0
	Propofol	0	0	0	0	0	0
	Physical method	0	0	0	1 (3.7%)	0	0
	Freezing	0	0	2 (8.3%)	0	0	0
	Removal from Water	0	0	0	0	0	1 (7.7%)
	Other	0	0	0	0	0	0
		**Taxa**					
**2**^st^ **step** **method**		**Bivalve and gastropod** **mollusks**	**Cephalopod** **mollusks**	**Cnidarians**	**Crustaceans**	**Echinoderms**	**Horseshoe** **crabs**
Immersion	MS-222	0	0	0	0	0	0
	Clove oil/Eugenol/AquiS	0	0	0	0	0	0
	Magnesium chloride or magnesium sulfate	0	3 (13.0%)	2 (8.3%)	0	0	0
	1–10% ethanol	0	1 (4.3%)	0	0	1 (4.8%)	0
	>50% ethanol	0	0	3 (12.5%)	2 (7.4%)	0	1 (7.7%)
	Formalin	1 (8.3%)	0	5 (20.8%)	2 (7.4%)	2 (9.5%)	1 (7.7%)
	2-PE	0	0	0	0/27	0	0
Injection	KCl	0	2 (8.7%)	0	4 (14.8%)	0	2 (15.4%)
	Pentobarbital	0	4 (17.4%)	0	4 (14.8%)	0	4 (30.8%)
	Lidocaine	0	0	0	1 (3.7%)	0	0
	Propofol	0	0	0	0	0	0
	Physical method	1 (8.3%)	11 (47.8%)	1 (4.2%)	7 (25.9%)	5 (23.8%)	2 (15.4%)
	Freezing	7 (58.3%)	4 (17.4%)	11 (45.8%)	10 (37.0%)	11 (52.4%)	2 (15.4%)
	Removal from water	1 (8.3%)	0	1 (4.2%)	1 (3.7%)	1 (4.8%)	0
	Other	0	0	0	1 (3.7%)	0	0

Respondents were permitted to select all options that apply so the total count of percentages are >100%.

To be considered euthanasia, an animal should be quickly rendered non-responsive and the method should minimize stress, be reliable, reproducible, and irreversible (44). A two-step approach is recommended for the euthanasia of aquatic invertebrates by the American Veterinary Medical Association (43). The first step should render the animal non-responsive and can include immersion in anesthetics such as magnesium salts (MgCl_2_ or MgSO_4_), clove oil, eugenol, or ethanol (1-10%). Injections of potassium chloride in direct proximity to the ventral nerve chord or injectable anesthetics can be used in crustaceans (45, 46). The second step should be unsurvivable and include physical or chemical destruction of the brain or major ganglia. Acceptable options for the second step include immersion in 70% alcohol, 10% formalin, or physical methods such as pithing, freezing, boiling, or sharp dissection. Methods that are unacceptable as a first or solo step include removal from the water to die by desiccation and hypoxia, freezing, or immersion in caustic chemicals (such as tissue fixative or 70% ethanol) (43).

## Opportunity 2: Development of less invasive sampling methods for research and diagnostic procedures

Research protocols and diagnostic procedures in aquatic invertebrates often involve terminally collected samples which may not be sustainable considering population declines in many invertebrate species. As of 2021, the International Union for Conservation of Nature (IUCN) lists 1,661 invertebrate species as critically endangered or endangered and an additional 1,326 species as threatened (47). Furthermore, lethal sampling may become unacceptable due to changing public attitudes and increasing animal welfare concerns by the scientific and animal health communities.

In animal research, scientists are obligated to use the 3Rs (replacement, reduction, and refinement) as a framework for the humane treatment of animals. The 3Rs were originally developed in 1959 by Russel and Burch (48) to improve laboratory animal welfare but are generally applicable to any situation where animals and humans interact. Replacement refers to replacing the use of animals; this can include the use of *in vitro* and *in silico* models. Reduction refers to the use of appropriate experimental design to appropriately power a study and optimize the number of animals used, as well as the data collected from each animal. Refinement refers to minimization of the pain, suffering, distress, and harm experienced by research animals (49).

We support application of the 3Rs principles across the animal kingdom. In various research settings and for diagnostic testing, lethal sampling techniques can be replaced with non-lethal procedures, including collection of hemolymph, coelomic fluid, or tissue biopsies. Current guidelines for blood collection in mammals limit removal to 10% of the total circulating blood volume (50), but very few analogous recommendations exist for invertebrates. Hemolymph and coelomic fluid removal should be limited to the minimum amount necessary, and perhaps no more than 10% of the circulating volume until safe guidelines can be established through research. Tissue biopsies should also be kept to the minimum practical size needed to fulfill sampling objectives. Only a few milligrams of tissue are necessary for conservation genetics and other molecular testing. Non-lethal sampling has been performed in sponges, corals, crustaceans, insects, echinoderms, and mollusks (51). Examples of non-lethal procedures include *in vivo* solid phase microextraction using a fiber inserted near the mouth of the animal to evaluate plastic contaminants in corals and bivalves (52) and the use of dragonfly fecal pellets and shed exoskeletons for DNA extraction (53). Further refinement can include the use of anesthetics and analgesics for invasive procedures. Handling techniques can be evaluated and improved to minimize stress and harm. If non-lethal sampling is performed but animals must be permanently removed from the wild, a plan to provide life-long care presents an opportunity for placement in educational or display settings. For example, if planned in advance, disposition to public aquaria may be an option for some non-releasable invertebrates, depending on capacity and institutional collection plans.

In cases where invasive sampling cannot be avoided, sharing samples with other researchers can reduce the need for additional specimen collection (54). If lethal sampling is required, aquatic invertebrates should be euthanized prior to sampling. Due to concerns over sample quality, invertebrates are often terminally sampled without methods rendering them non-responsive prior to sampling. However, several studies have demonstrated that high quality samples can still be obtained from euthanized animals. High quality RNA was successfully extracted from sea stars immersed in MgCl_2_ prior to sampling (55) and jellyfish immersed in MgCl_2_ provided useful samples for NMR-based metabolomics (56).

## Opportunity 3: Use of humane slaughter methods for aquatic invertebrates

Aquatic invertebrates including non-cephalopod mollusks (e.g., bivalves and gastropods), crustaceans, cephalopod mollusks, jellyfish, sea cucumbers, and sea urchins are commonly consumed by humans with 41 million tons captured or cultured in 2018 (57). While euthanasia methods are published for many of these taxa, there is a lack of peer-reviewed literature evaluating humane stunning and slaughter methods. The only taxon with published information on humane slaughter are decapod crustaceans (58). Surprisingly, while cephalopods are the focus of much research on sentience and pain perception, no published article could be found on appropriate slaughter techniques for this taxon, as of April 2022. Methods for cephalopod slaughter include decapitation without prior stunning (59), “clubbing, slicing the brain, reversing the mantle, and asphyxiation in a suspended net bag”, none of which are considered to be humane (29).

The debate on humane slaughter methods for decapod crustaceans started in the 1950s with publications by Baker and Gunter (60, 61). There is contradictory evidence on whether slowly warming live animals or placing live animals in boiling water is humane (62, 63), but boiling lobsters alive has been banned in New Zealand (64), Norway (26), Switzerland (65), and certain parts of Italy (66). Ice slurries and electrical shock may paralyze crustaceans, but neural circuits still remain intact and functional so these methods are likely best used after rendering the animal insensible (67). Based on the available scientific evidence, all animals should have their nerve ganglia destroyed prior to cooking to prevent any potential suffering (60, 68). A commercially available stunning device, the CrustaStun (Mitchell & Cooper, Uckfield, England) that is recommended by the Royal Society for the Prevention of Cruelty to Animals (RSCPA), can be used to stun crustaceans prior to boiling and has been shown to arrest nervous activity after use (69).

Regardless of the method for slaughter chosen, stress should be minimized throughout the supply chain and animals should be killed quickly to avoid unnecessary suffering and pain. More evidence-based and species-appropriate methods are needed for practical humane slaughter of aquatic invertebrates, particularly for cephalopod mollusks.

## Opportunity 4: Reduce the impact of invasive procedures in aquaculture and fisheries

Crustaceans have been shown to experience stress and likely have the capacity to feel pain, which should be considered during processes from collection to slaughter. Industry practices that might be adjusted to minimize stress include decreasing the trawling duration, live transport duration, and handling needs (70). Additional good practice recommendations include maintenance of the animals' thermal preference zone, provision of good water quality, and allowance of recovery periods (71). Anesthetics can also be used to decrease stress throughout the supply chain from collection to slaughter. Isobutanol, a food safe anesthetic, reduced ammonia concentrations and mortalities during live transport of tropical spiny lobsters (*Panulirus* spp.) (72).

Crabs in fisheries worldwide have their claws manually removed followed by release back into the water. Live declawing is performed with brown crabs (*Cancer pagurus*) (73), stone crabs (*Menippe* spp.) (74), and fiddler crabs (*Uca tangeri*) (75). While crustaceans do autotomize claws naturally, manual declawing is more stressful and causes significantly higher mortality than natural autotomy (73). This practice is often considered better than whole crab landing, based on the assumption that these animals survive and regenerate their claws, while remaining in the fishery. However, mortality was >60% in stone crabs with both claws removed (76) and regenerated claws only comprised 3% of legal stone crab landings (77), indicating that this practice is little or no more sustainable than whole crab harvest. Crabs that survive declawing show decreased feeding (74, 78) and decreased reproductive fitness (75, 79). Based on animal welfare concerns and negligible population benefits, declawing may not be preferable over humane harvest and slaughter.

Brown crabs are often transported alive, and mechanisms are needed to prevent them from damaging each other during transport. In the Irish fishery, they undergo a process known as nicking, which involves cutting the ligament under the dactylus of the claw since their claw shape makes traditional banding used in other crustaceans challenging (80). Nicking results in hemolymph loss, risk of infection, inability to molt, and increased mortality (81–83). Nicked crabs had higher hemolymph glucose, lactate, and refractive index, indicating they experienced increased stress (80). In the Norwegian fishery, the crabs are not nicked and are instead transported dry (84). However, emersion can also result in welfare issues, particularly at higher temperatures (85). Adapted banding techniques [e.g., Elastrator (castrator) rings combined with a wooden dowel through the claw] could be considered (86). Finding a solution that balances crab welfare with the needs of the fisheries offers an opportunity for research.

In shrimp aquaculture, eyestalk ablation is performed to induce female broodstock to spawn, since the eyestalks are a source of vitellogenesis-inhibiting hormone (VIH) which is a negative regulator of crustacean reproduction (87). Following eyestalk ablation, shrimp exhibit stress-related behaviors including erratic and spiral swimming, rubbing, and tail flicking, which are prevented by topical anesthetic application (88, 89). Beyond the stress and potential pain caused by handling and the procedure, eyestalk ablation can also impact the immune system of shrimp (90). Non-ablated broodstock females appear to perform at a similar level as ablated females, with larvae that are more resilient to typical pathogens and environmental stress (91, 92). As eyestalk ablation carries negative health and welfare consequences, evaluation of alternatives could be beneficial. Switching from a 1:1 ratio of females to males to a 1:2 ratio improves performance without ablation (91) and a single injection of anti-GIH monoclonal antibody was shown to have similar performance to eyestalk ablation (93).

## Conclusions

Aquatic animal health professionals believe that aquatic invertebrates, especially cephalopods, can feel pain. However, <50% have used analgesia during invasive procedures with aquatic invertebrates, likely due to a dearth of well described effective options. This highlights the need for more research on appropriate anesthetic and analgesic options for aquatic invertebrates. While the discussion of pain perception in invertebrates is important, the ability to feel pain is not a prerequisite for promoting positive animal welfare in aquatic invertebrates. Many cost- and time-effective opportunities for the improvement of aquatic invertebrate welfare exist and can be appropriate in various settings. We advocate the use of these advancements and further investigations in this underrepresented but important field of animal welfare.

## Data availability statement

The raw data supporting the conclusions of this article will be made available by the authors, without undue reservation.

## Author contributions

SW and NS conceptualized the presented idea. All authors contributed to the article and approved the submitted version.

## Conflict of interest

Author AN is employed by ZooQuatic Laboratory, LLC. The remaining authors declare that the research was conducted in the absence of any commercial or financial relationships that could be construed as a potential conflict of interest.

## Publisher's note

All claims expressed in this article are solely those of the authors and do not necessarily represent those of their affiliated organizations, or those of the publisher, the editors and the reviewers. Any product that may be evaluated in this article, or claim that may be made by its manufacturer, is not guaranteed or endorsed by the publisher.
